# Supplementary figures and images for: Minigene Splice Assays Allow Pathogenicity Reclassification of RPE65 Variants of Uncertain Significance
Source: Genes (Basel). 2025 Aug 28;16(9):1022. doi: 10.3390/genes16091022 (PMC12469365; doi:10.3390/genes16091022)

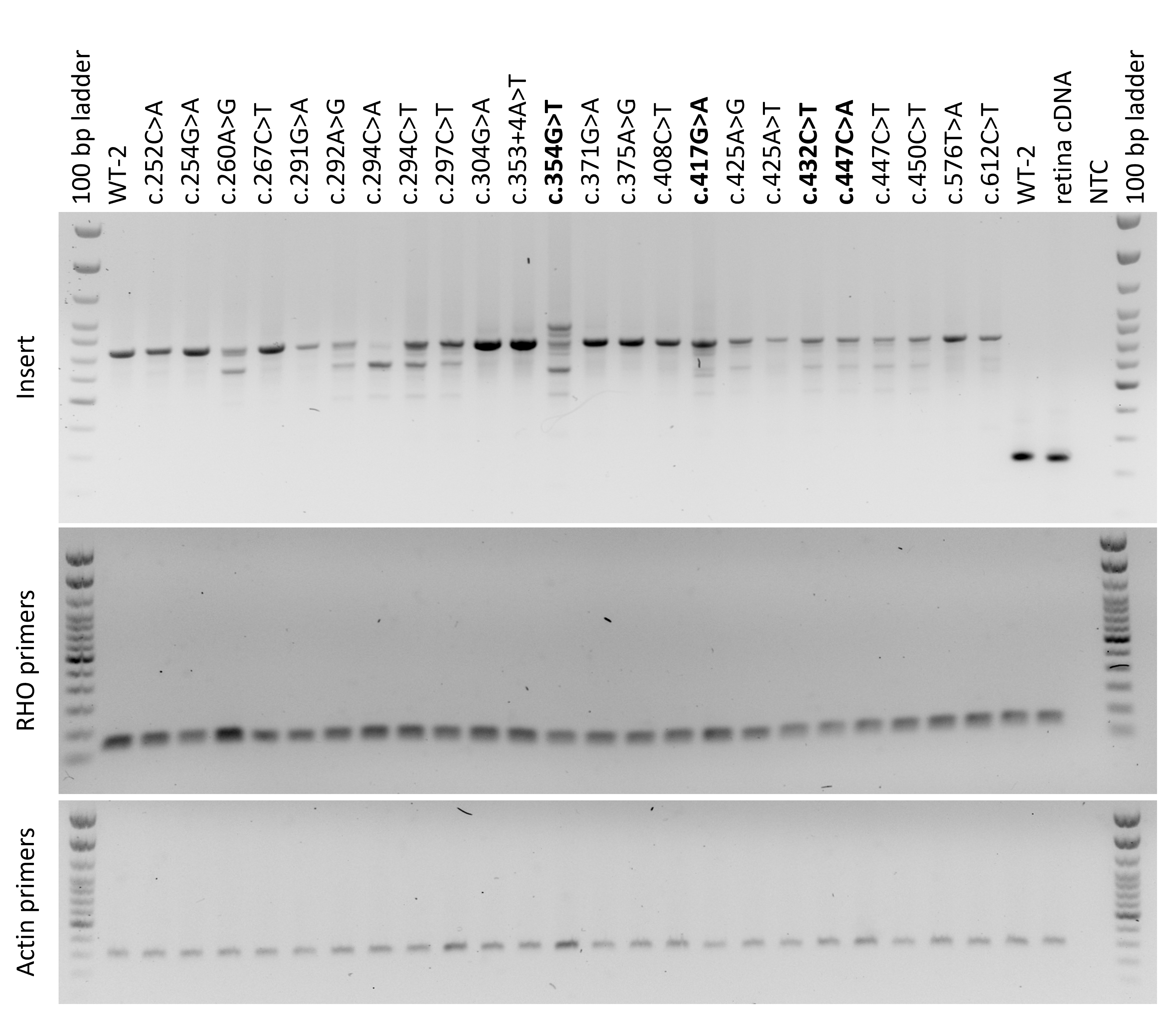

Supplement: Supplementary file 1 [file genes-16-01022-s001.zip › Supplemental figure 1.tif]

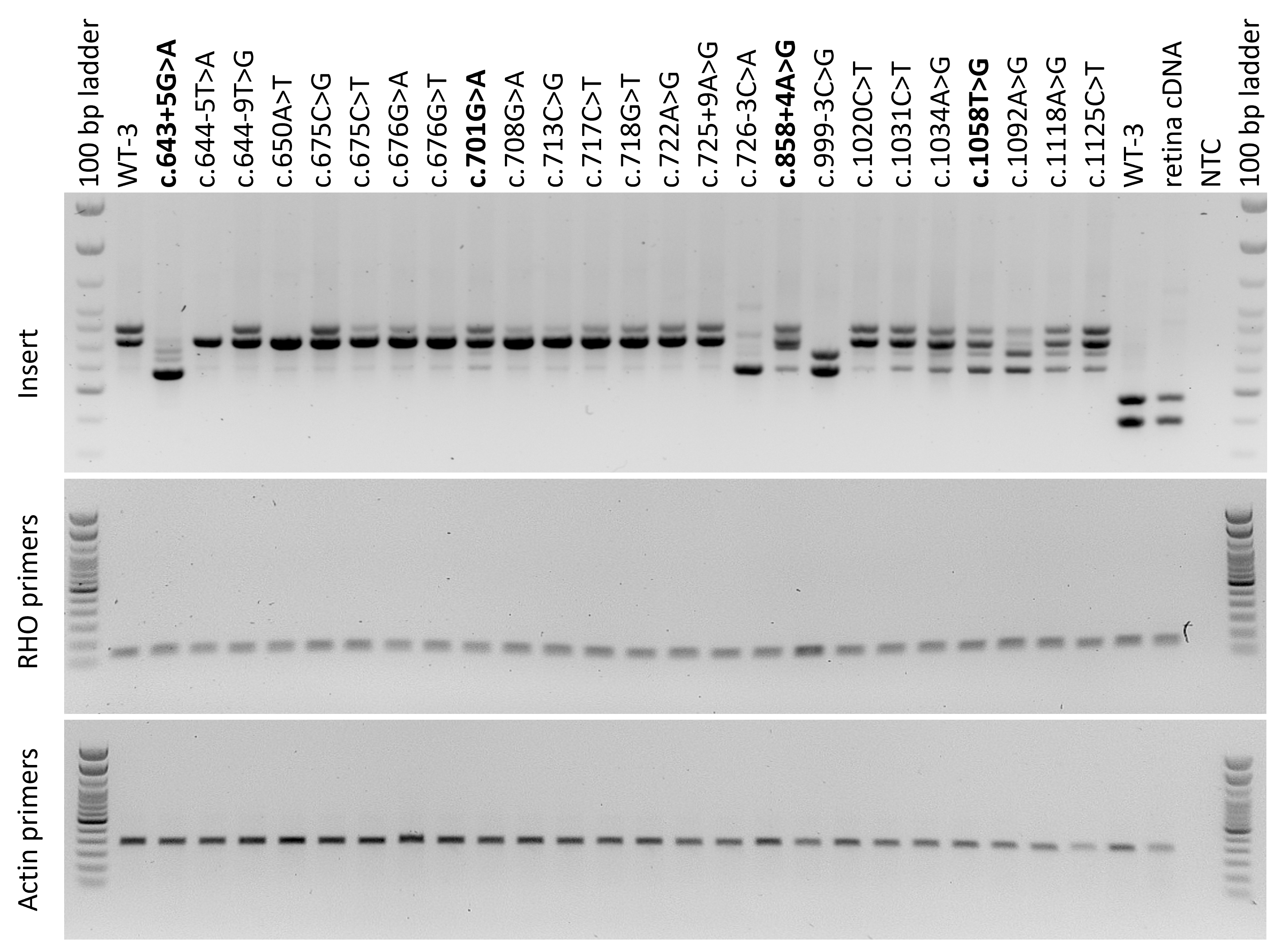

Supplement: Supplementary file 1 [file genes-16-01022-s001.zip › Supplemental figure 2.tif]

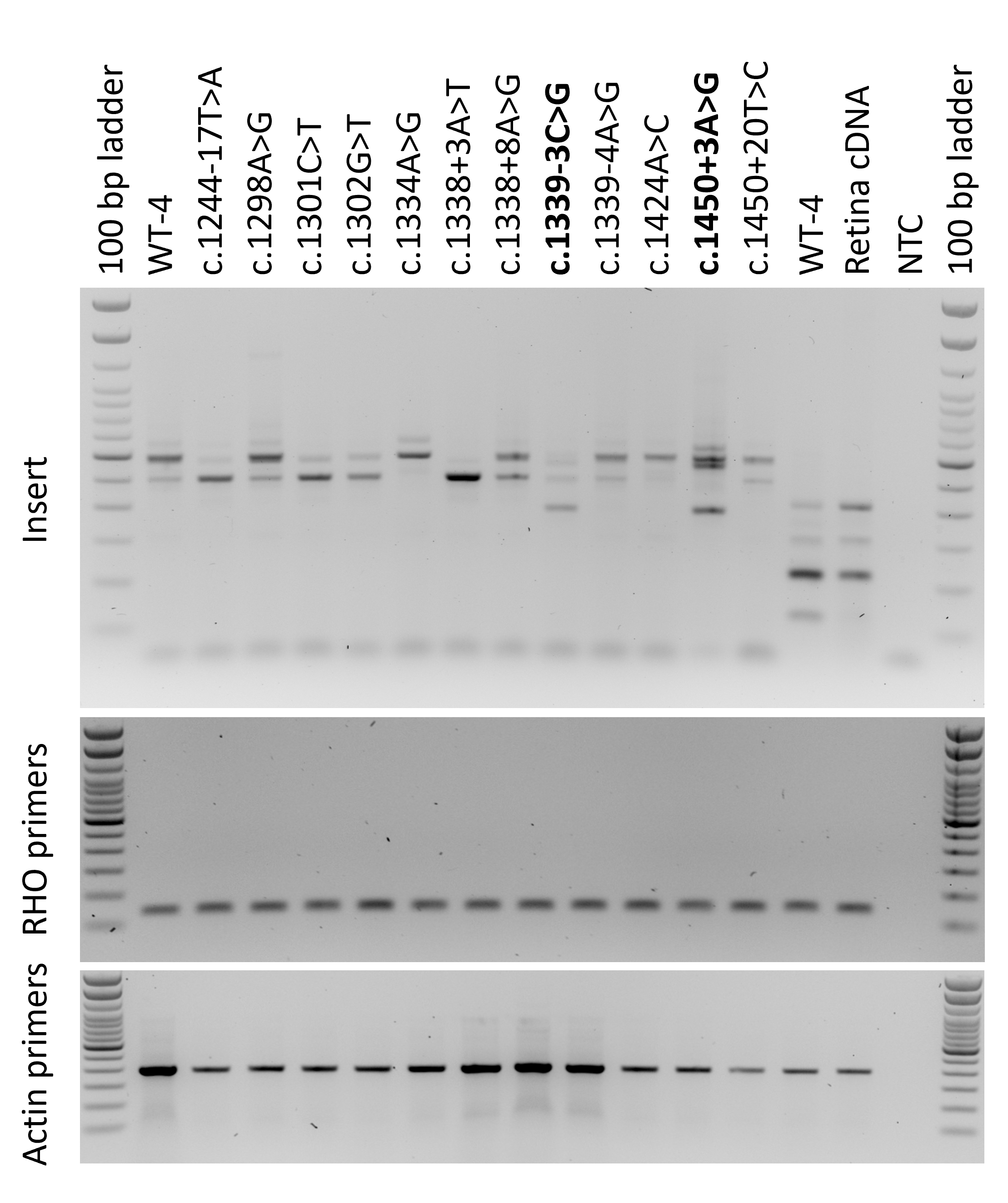

Supplement: Supplementary file 1 [file genes-16-01022-s001.zip › Supplemental figure 3.tif]

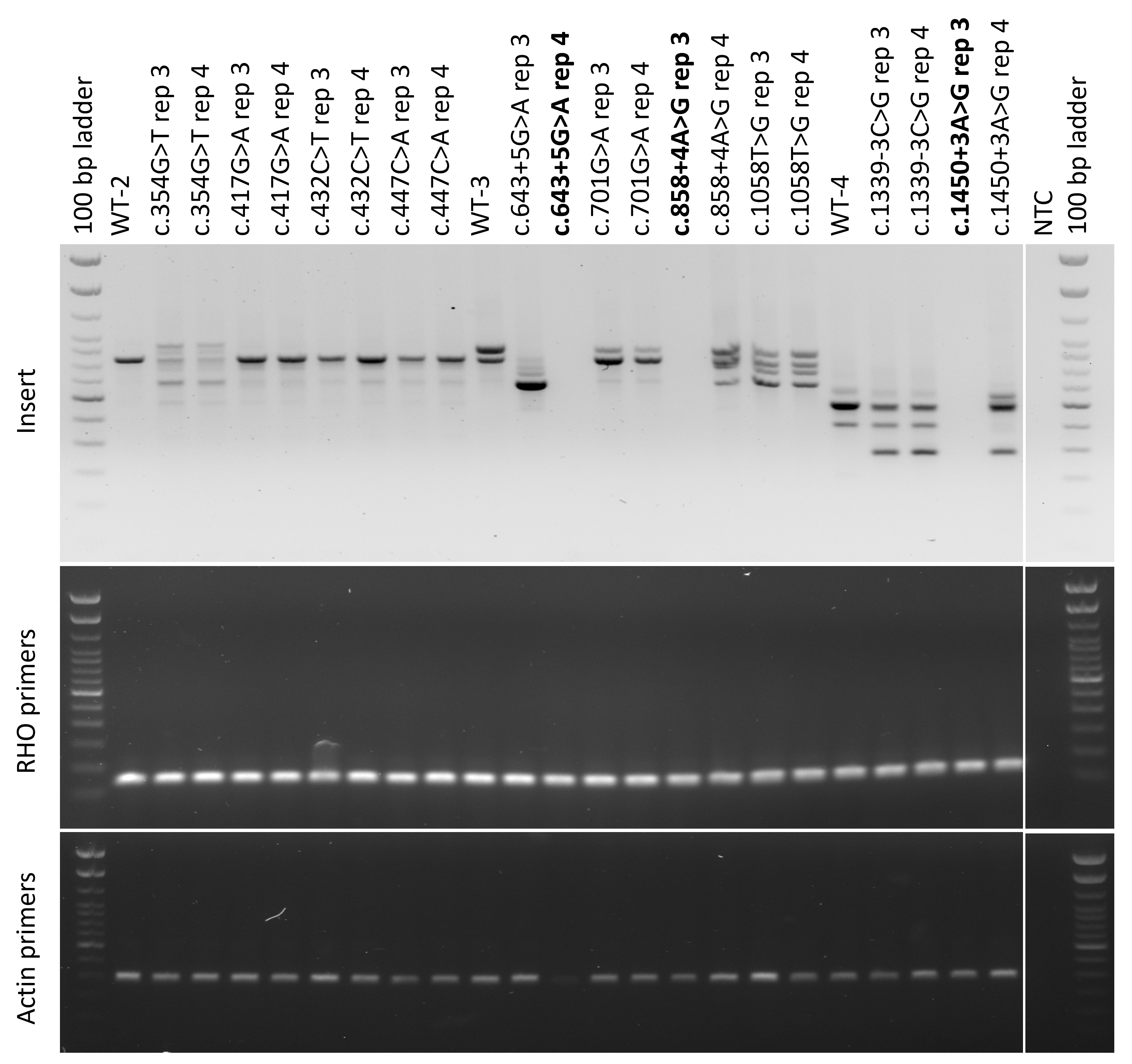

Supplement: Supplementary file 1 [file genes-16-01022-s001.zip › Supplemental figure 4.tif]
